# Single-Layer and Double-Layer Filtration Materials Based on Polyvinylidene Fluoride-Co-hexafluoropropylene Nanofibers Coated on Melamine Microfibers

**DOI:** 10.1021/acsanm.3c02592

**Published:** 2023-08-22

**Authors:** Tilen Potisk, Maja Remškar, Luka Pirker, Gregor Filipič, Igor Mihelič, Marjan Ješelnik, Urban Čoko, Miha Ravnik

**Affiliations:** † Laboratory for Molecular Modeling, National Institute of Chemistry, SI-1001 Ljubljana, Slovenia; ‡ Faculty of Mathematics and Physics, University of Ljubljana, SI-1001 Ljubljana, Slovenia; § Jožef Stefan Institute, SI-1000 Ljubljana, Slovenia; ¶ J. Heyrovsky Institute of Physical Chemistry, Czech Academy of Sciences, 182 23 Prague 8, Czech Republic; ∥ MELAMIN d.d., SI-1330 Kočevje, Slovenia

**Keywords:** nanofibers, aerosols, filtration, simulations, modeling

## Abstract

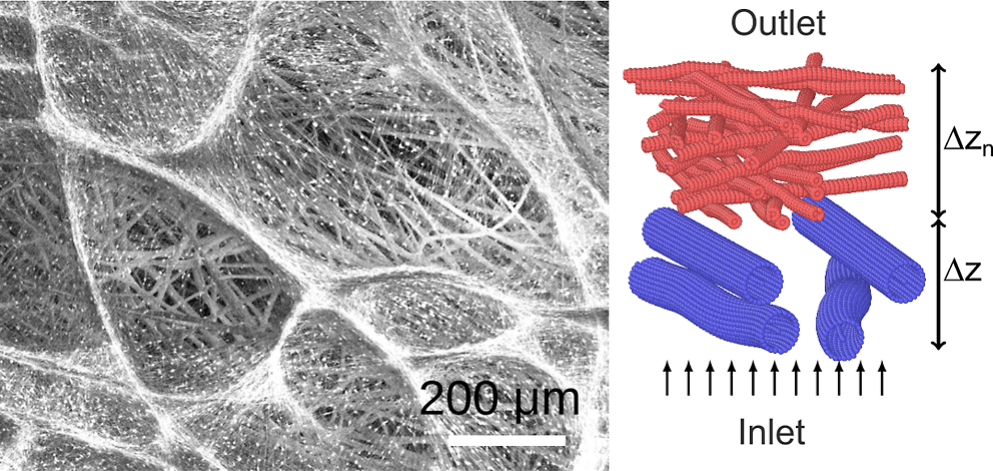

In this work, we demonstrate selected optimization changes in the simple design of filtration masks to increase particle removal efficiency (PRE) and filter quality factor by combining experiments and numerical modeling. In particular, we focus on single-layer filters fabricated from uniform thickness fibers and double-layer filters consisting of a layer of highly permeable thick fibers as a support and a thin layer of filtering electrospun nanofibers. For single-layer filters, we demonstrate performance improvement in terms of the quality factor by optimizing the geometry of the composition. We show significantly better PRE performance for filters composed of micrometer-sized fibers covered by a thin layer of electrospun nanofibers. This work is motivated and carried out in collaboration with a targeted industrial development of selected melamine-based filter nano- and micromaterials.

## 1 Introduction

Inhalation of aerosols containing air pollutants such as heavy metals,^1,2^ nanoparticles,^3^ and particulate matter (PM_2.5_ and PM_10_)^4^ can lead to cardiovascular^5^ and pulmonary^2^ diseases, a higher risk of lung cancer, neurological and psychiatric problems, higher mortality rates, reproductive and developmental problems,^2^ and a generally shorter life expectancy.^4,6^ Highly infectious diseases that spread via respiratory droplets and aerosols can cause severe respiratory infections,^7^ leading to a pandemic such as Covid-19. In the case of airborne diseases, air filters, masks, and barriers have proven effective in dramatically slowing down the spread of droplets and aerosols.^8,9^ They should also be effective in dry conditions when the size of respiratory droplets decreases and the smallest droplets become nanoscopic. Such small droplets remain in the atmosphere for a long time and contribute to aerosol-transmitted infections.^10^ It is therefore of utmost importance to develop filters with a high particle removal efficiency (PRE) that are also comfortable to wear for extended periods of time.

Several experimental studies have evaluated the performance of fibrous filters.^11–15^ The typical filters of commercially available masks consist of layers with fiber sizes in the micrometer range, usually made of polypropylene. Their filtration performance depends on the standard used in their manufacture, but most high-quality masks provide a high level of protection by filtering the majority of aerosols.^11^ Equivalent or better filtration performance was observed with nanofiber filters. This is due to their higher surface-to-volume ratio, electrostatic properties, and greater slip effects around the fibers, resulting in a lower pressure drop.^16–18^ Nanofibers can be produced by various methods, such as wet spinning,^19^ dry or melt spinning,^20^ template synthesis,^21^ solution blow spinning,^22^ force spinning,^23^ and electrospinning,^24^ in which small diameter and highly porous fibers are deposited on a porous and mechanically more stable structure. The nanofibers can be further functionalized, making the mask multifunctional without compromising filtration efficiency or breathability.^25^ In addition, electrospun nanofibers can provide novel functionalities, e.g., stimuli-responsive materials for activated disinfection,^26,27^ generation of electricity for self-powered wearable electronics,^28^ and increase of the hydrophobicity, charge, and its retention.^29^

With the outbreak of the Covid 19 pandemic, various experimental procedures have been proposed to test the filtration performance of masks. Typically, NaCl, KCl, or aerosols produced by live subjects are used in combination with a particle counter to assess the filtration efficiency of masks.^30–32^

The widespread use of disposable respiratory masks during the Covid-19 pandemic led to an increased environmental burden^33–35^ and prompted a worldwide search for mask recycling and reuse.^36,37^ Thermal disinfection is one of the possible solutions, but it usually leads to reduced filtration efficiency due to thermal decomposition of fibers or loss of surface charge.^12,38^ Therefore, the materials must be thermally stable. In addition, respiratory masks must be sufficiently flame-resistant,^39–41^ which has become an important issue in oxygen-rich environments such as hospital intensive care units^42^ and surgical operating theaters.^43^ One promising material is melamine, a nitrogen-based compound with various applications that is also a known fire retardant due to the release of nitrogen gas during combustion.^44^ When combined with formaldehyde, it produces a thermoplastic compound that can be formed into a fibrous shape.^45^ As formaldehyde is only used as a precursor in the formation of melamine fibers, it is not present in free form in the final product, which could lead to health risks.^46^ In addition, the thermal and chemical stabilities of melamine could allow masks to be sterilized without the loss of PRE.

Several simulation studies have been conducted on the subject of increasing the filtration performance of mechanical filters. It has been shown that filters with staggered fiber layouts perform better than filters with parallel fiber orientations.^47^ Electrostatic effects due to the charged particles and fibers or external electric fields were also shown to dramatically increase the particle removal efficiency while having a small pressure drop.^48,49^ The optimal distribution of fibers within the filter was also investigated.^50^ It was found that a uniform distribution of the fibers in terms of the solid volume fraction provides the lowest pressure drop. In addition, placing thinner fibers near the inlet and larger fibers near the outlet provides the best particle removal efficiency. The aforementioned study, however, focused only on micron-sized fibers. Moreover, the model assumed a parallel orientation of fibers, which limits its application to such filters. The distribution of fiber orientation has a great influence on the filtration performance, as demonstrated experimentally^51^ and theoretically.^52–54^ Some works also considered the facial anatomy, which significantly affects the overall airflow through the fibers,^55^ and the effects of breeze on the spread of saliva particles.^56,57^ Typically, the methods involved consist of solving the Navier–Stokes equations^48,49^ or using the Lattice-Boltzmann Method (LBM)^47,50,58,59^ to describe the gas flow around the filter fibers, coupled with the Langevin equation for the dynamics of the aerosols. To the best of our knowledge, no simulations have yet been performed to study the effects of the simple expansion of a fibrous filter along the flow direction. Moreover, numerical studies on the optimization of double-layer filters containing random 3D-oriented micrometer-sized fibers and nanofibers are lacking.

In this study, we present both experimental and modeling optimizations of melamine fiber-based filtration materials for face masks. This effort was inspired and conducted in response to specific industrial developments amid the Covid-19 pandemic. The work is carried out in two directions: to explore the main geometrical composition of the single fiber filtration and the two-layer filter based on a thick support layer and an overlying (electrospun) thin film. The motivation behind the two-layer filter is to use nanofibers on top of highly porous sublayers to obtain a highly efficient filter with a low pressure drop. In Section 2, we describe the experimental setup for performing the PRE measurement and fabrication of thin electrospun polyvinylidene fluoride (PVDF-HFP) fibers, the simulation system, and the corresponding computational model. Section 3 presents the results including the analysis of pressure drop, particle removal efficiency, and quality factor of various filter designs. More generally, this work contributes to the optimization and development of nanostructured air filter materials, including for face masks.

## 2 Materials and Methods

### 2.1 Filter Material Production

**Figure 1:**
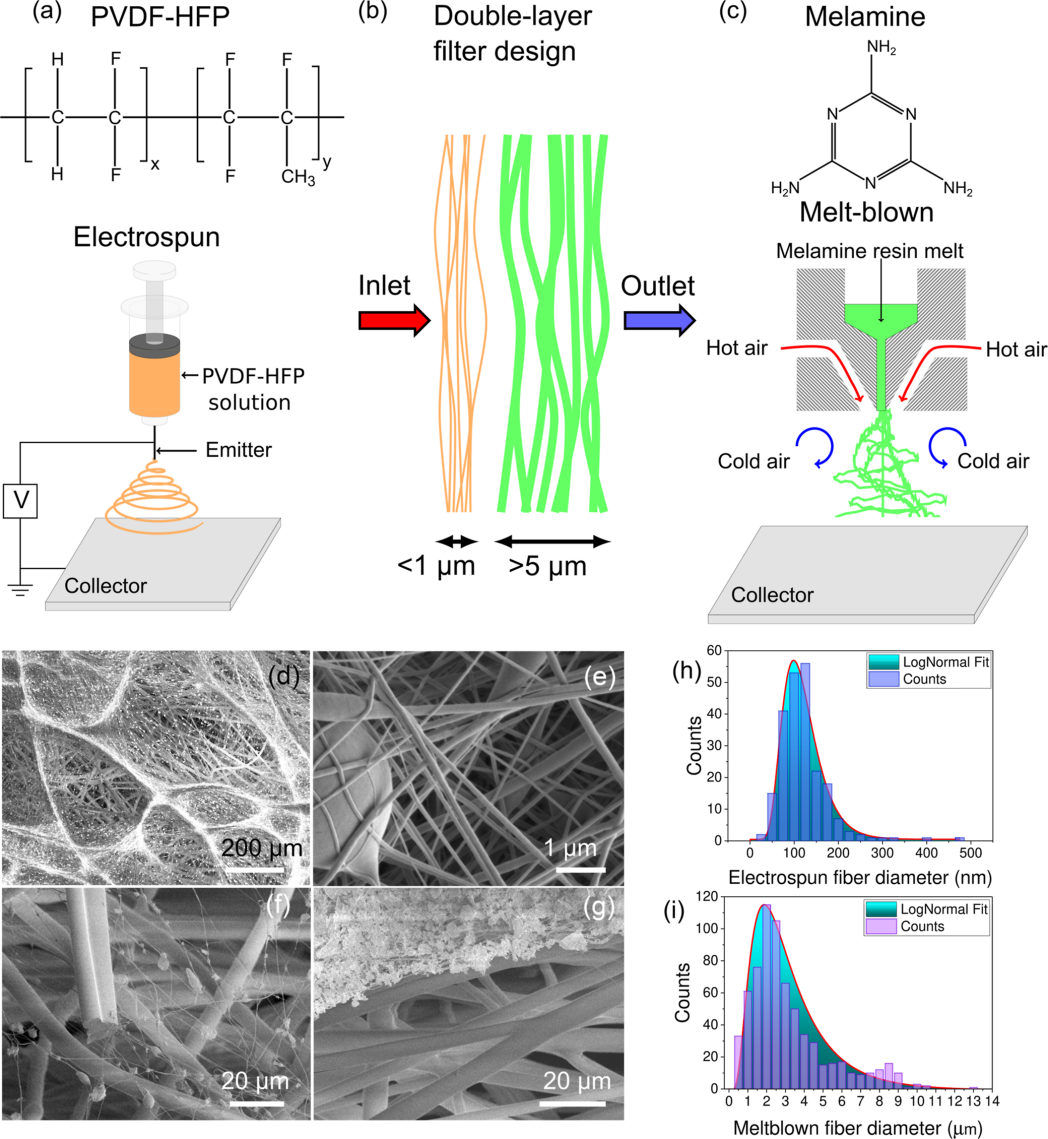
(a) Schematic diagram of the production of PVDF-HFP nanofibers by electrospinning. (b) Schematic of the double-layer filter design with a thin layer of nanofibers and a layer of micrometer-sized melamine fibers. (c) Schematic of melamine fiber fabrication using melt-blown process. SEM images of (d) PVDF-HFP nanofibers spun on melamine microfibers; (e) PVDF-HFP nanofibers with PVDF-HFP polymer bead; (f) low density of PVDF-HFP fibers after particles filtration; and (g) exposed melamine fibers (lower part) after removal of PVDF-HFP fibers with captured particles (upper part). (h) Size distribution of PVDF-HFP nanofibers. (i) Size distribution of melamine micron-sized fibers.

Single-layer filter materials are based on melamine fibers with the industrial name smartMELAMINE,^60,61^ produced by the melt-blown process from melamine resins, see Figure 1c for a schematic. The nonwoven melamine fibers combine the properties of raw materials with the advantages of a melt-blown. They belong to the class of high-performance materials that do not burn, do not shrink, and do not melt. They remain stable up to 240 °C. The formaldehyde content in these fibers is below the detection limit (EN ISO 14184–1). The fibers are insoluble in water and odorless. Melamine is UV and chemically resistant, is a thermal and acoustic insulator, and is also well suited for filtration due to the very fine fiber diameters, which range from 1–15 μm (the average diameter is 2.4 μm, see Figure 1i).

Two-layer filter materials are made by applying polyvinylidene fluoride-*co*-hexafluoropropylene (PVDF-HFP) as a top layer on the melamine fibers; see Figure 1b for a scheme of the double-layer filter design. Polyvinylidene fluoride (PVDF) has some favorable properties for particle filtration and use in face masks: it is chemically inert, i.e., it does not react or decompose during use; it is resistant to abrasion damage, allowing respiratory mask handling without damage; it is stable under UV irradiation and up to 150 °C, so the mask can be worn in the sun; and it is hydrophobic, meaning the moisture from the breath would not wet it and reduce the filtration performance.^62,63^ In addition, electrospinning triggers a poling process and allows PVDF to be piezoelectric. The polarized polymer could provide higher filtration efficiency due to the electrostatic interaction with the nanoparticles, and among the known polymers, PVDF exhibits one of the strongest piezoelectric effects.^64^ The addition of hexafluoropropylene (HFP) to PVDF further improves the properties of the copolymer: the hydrophobic effect as well as the mechanical strength and flexibility of the copolymer^65^ are increased Moreover, the addition of HFP helps to stabilize the β-phase of PVDF, thus increasing the piezoelectric properties of the copolymer.^66^ The high chemical and thermal stability of PVDF-HFP also allows it to be used in medicine, e.g., as biocompatible membranes and implants.^67^

To prepare the nanofiber overlayer, a solution of 18 wt % PVDF-HFP (Sigma-Aldrich, Merck d.o.o., Slovenia, Europe) in n,n-dimethylformamide (DMF, Carlo Erba Reagents GmbH, Germany) was stirred for several hours at 60 °C and then electrospun onto the thick fiber melamine base layer; see Figure 1a for a schematic. The PVDF-HFP solution was placed in a plastic syringe fitted with a 0.8 mm diameter needle. A syringe pump (Razel R99-E, Razel Scientific Instruments, USA) was used to feed the solution into the needle at a flow rate of 0.17 mL h^–1^. The positive output lead of a high-voltage power supply (HVG-P60-R-EU, Linari Engineering, Italy, Europe) was attached to the needle. An electrically grounded aluminum foil served as the collector. The applied voltage between the collector and the needle was 15 kV, and the collector-to-needle distance was 20 cm. The supporting melamine material was placed on top of the aluminum foil; see Figure 1d–g for scanning electron microscopy (SEM) images of PVDF-HFP-melamine structures. During electrospinning, not only fibers but also polymer beads were created. These are a byproduct of the experimental setup where the conditions, i.e., temperature and homogeneous polymer precursor flow, could not be perfectly controlled to produce pure fibers. However, the concentration of the polymer beads is sufficiently low to not affect the filtration efficiency or invalidate the results of the simulations. The electrospun PVDF-HFP nanofibers, several layers thick, formed on top of the melamine fibers, Figure 1d,e. A thinner layer of PVDF-HFP fibers formed between the topmost melamine fibers, as shown in Figure 1f. The thin layer is only a few PVDF-HFP fibers thick, with a thickness below 1 μm (the average diameter of the PVDF-HFP nanofibers is 120 nm, see Figure 1h).

### 2.2 Filter Efficiency Measurements

The filtration efficiency of melamine fibers and melamine fibers with electrospun PVDF-HFP nanofibers was measured using a Scanning Mobility Particle Sizer (SMPS model 3080 L85; TSI Co., Shoreview, MN, USA) equipped with a desiccator, a soft X-ray neutralizer, a long differential mobility analyzer (DMA), and a water condensation particle counter (WCPC; model 3785; TSI).^11^ The electrical mobility diameter of the counted nanoparticles ranges from 13 to 572.5 nm, and the airflow at the inlet of the SMPS was 4.1 L min^–1^. The powder composed of silica (69%–77%), aluminum oxide (8%–14%), calcium oxide (2.5%–5.5%), and other mineral oxides (in a total concentration of up to 5%), conforming to the ISO 12103–1 A1 aerosol standard, was dispersed in the sampling chamber during measurements using a Topas SAG 410 aerosol generator with the following parameters: *p*_*inlet*_ = 1 bar, feed rate 3.0%, and preparation 3/7.

The overall PRE is calculated as the normalized difference between the total number of transmitted particles without (*T*_OFF_) and those with (*T*_ON_) filters
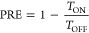
1

For *T*_OFF_, five measurements without the use of the filter material are averaged, whereas the third measurement with the use of the filter material is considered for *T*_ON_. After several sequential measurements (each 3 min long), the dust particles form a thick layer on the material, which increases the PRE of the material.

The PRE dependence on particle diameter (*d*_p_) is calculated as^11^
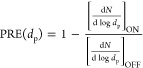
2where  is the normalized concentration of particles transmitted through the sample in the third measurement, and  is the normalized concentration of particles averaged over five measurements before the filter was applied.

### 2.3 Computational Filter Design

**Figure 2:**
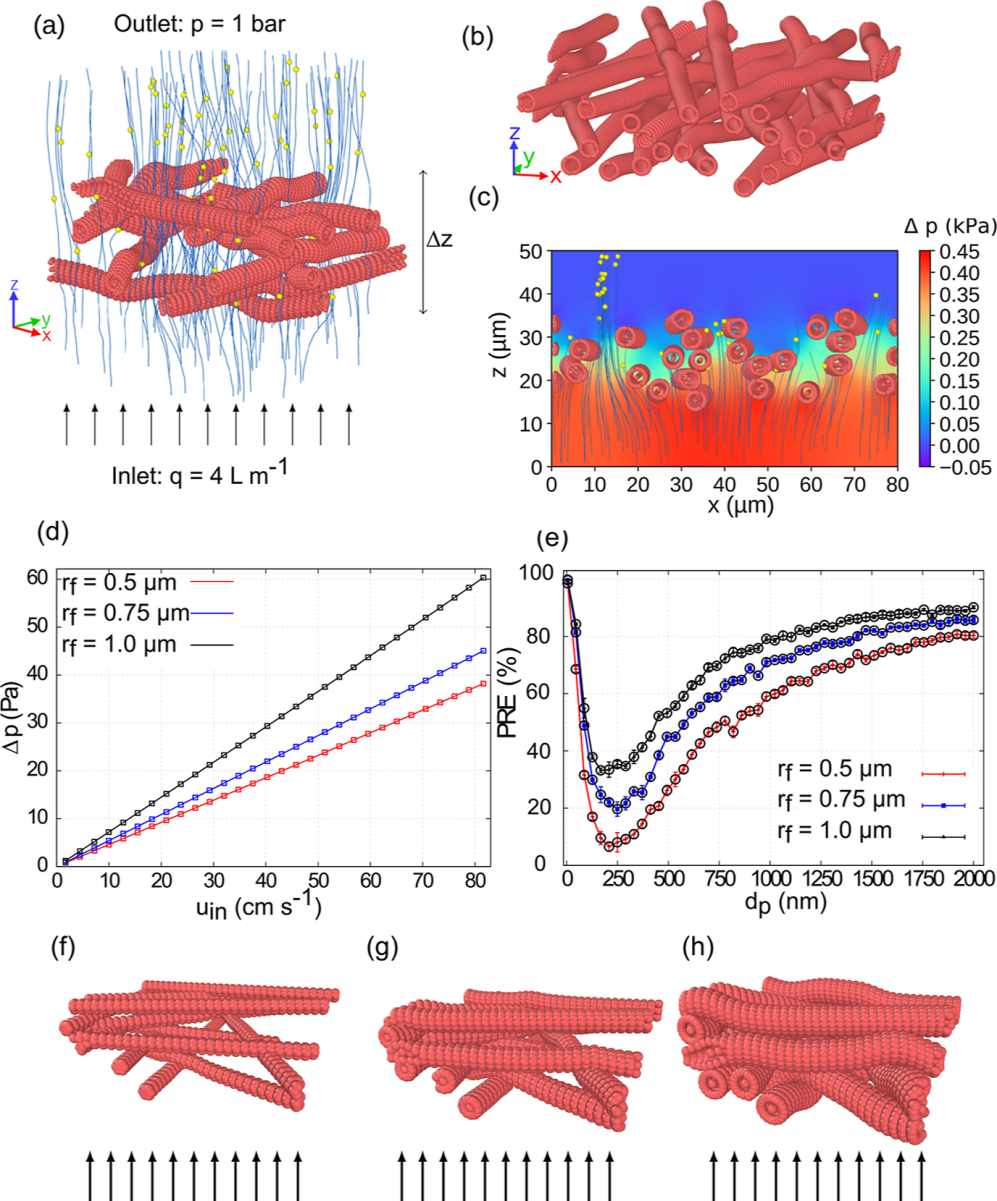
(a) Example of a computer-generated nonwoven mesh of fibers used in this study. At the inlet, a constant volume inflow of *q* = 4 L min^–1^ is specified, while at the outlet, we set the pressure to 1 bar. The thickness Δ*z* of the filter is defined as the difference in *z* coordinates of the lowest fiber surface bead and the highest fiber surface bead. This figure represents a unit cell of a filter that extends periodically in *x* and *y* dimensions and is open along the *z* axis. (b) Example of a filter with a small variation of the fiber orientation in the *xy* plane. (c) Corresponding pressure field and trajectories of aerosols in a filter with approximately parallel fibers in the *xz* plane, averaged in a thin slice around . (d) Pressure drop across a Δ*z* ≈ 9 μm thick filter material as a function of the inlet velocity at three different fiber radii. (e) PRE as a function of aerosol particle diameter *d*_*p*_ at three different fiber radii *r*_f_. Single-layer filters with fiber radii (f) 0.5, (g) 0.75, and (h) 1 μm used for pressure drop calculations.

In our simulations, the filter materials are considered to consist of a fluid part (air), filter fibers, and aerosols. The fibers are distributed randomly in the lateral (*x* and *y*) dimensions of the filter; see Figure 2a. The double-layer filters are constructed by placing a layer of thin nanofibers on top of a layer of thick fibers.

The filter topologies are created using the LAMMPS simulation package.^68^ We start by placing long, randomly oriented (in the *xy* plane) linear chains of beads in a simulation box with periodic boundary conditions in the *x* and *y* dimensions and open boundary conditions along the *z* axis. The beads are connected into chains by harmonic springs, described by the bond potential *V*_*b*_
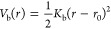
3where *K*_b_ is the bond constant, *r* is the distance between the connected beads, and *r*_0_ is the equilibrium bond distance. To keep the bond lengths approximately constant and equal to *r*_0_, the bond constant *K*_*b*_ is set to a large value,  with ε_*u*_ the energy unit and *r*_*u*_ the length unit which are set to 1. This requirement is simply to prevent large gaps from appearing in the generated fibers, which would lead to leakage of air into the fibers in our subsequent simulations and affect the calculated filter efficiency. In our work, we set *r*_0_ = 1.0 *r*_*u*_, which leads to satisfactory results in terms of the impermeability of the filter fibers. To prevent overlapping of the chains, a purely repulsive Weeks–Chandler–Anderson (WCA) potential^69^ is added between the beads
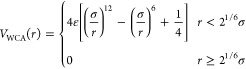
4where ε is the depth of the potential well and σ defines the scale of the force field. Here we found satisfactory results, i.e., no chain overlap, using ε = 1.0 ε_*u*_ and σ = 3.0 *r*_*f*_, where *r*_*f*_ is the dimensionless fiber radius. The structure is minimized using the conjugate gradient method,^70,71^ which iteratively adjusts the bead coordinates. The iteration is stopped when, in a given step, the potential energy changes by less than 10^–5^ in dimensionless units. We then generated the surfaces of the fibers by placing *N*_*b*_ = ⌊4*πr*_*f*_⌋ additional beads on a circle with the desired fiber radius *r*_*f*_ around each chain bead. The ⌊ ⌋ represents the floor function. Such a number of additional beads is again required to prevent leakage of air into the fibers. The circular arrangement of the beads can also be seen in the ribbed appearance of the fibers in Figure 2a. The porosity of the filter ε is related to the volume of the fibers *V*_*s*_ and the volume of the filter *V*_*f*_, , and is controlled by the initial distribution and number density of the polymer chains. We consider filters with porosities between 65% and 95%. An example of the computer-generated fiber network and the corresponding trajectories of the aerosols are shown in Figure 2a.

### 2.4 Numerical Method

To model the flow of air through the filter, we use the LBM.^72,73^ One of the advantages of LBM is the ease of implementing complicated boundary conditions. This makes it ideal for porous systems, such as fibrous filters. On the chosen length scales (nanometers to micrometers), it is also much faster than particle-based methods, where one tracks and solves dynamic equations for each gas particle. For simulating the 3D flow of air around filter fibers, we use the *D*3*Q*19 lattice, where particles are restricted to stream in 19 possible directions, **e**_*i*_ with *i* = 0, 1, ..., 18. For the collision term, the BGK operator^74^ with the relaxation parameter τ = 1.0 is chosen. For an overall scheme of the simulation method, see the flowchart in Figure S1. Since we will be dealing with fibers with thicknesses ranging from 100 nm to 1.5 μm, we choose for the lattice spacing Δ*x* = 100 nm for the small nanofibers and Δ*x* = 1 μm for the larger micron-sized fibers.

To model the dynamics of aerosols in air, we use the Langevin equation^75^
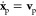
5
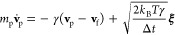
6where γ is the dissipation coefficient, *k*_B_ ≈ 1.38 × 10^–23^ J/K is the Boltzmann constant, *T* is the temperature,  is the mass of a given aerosol with density ρ_p_ and radius *r*_p_, **v**_p_ is the aerosol velocity, while ξ is a vector of uncorrelated Gaussian random variables with zero mean and unit variance.

For aerosols on a nanometer scale, the dissipation coefficient is the slip-corrected Stokes law for the flow around a spherical particle
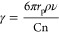
7where Cn denotes the Cunningham slip-correction factor^76,77^
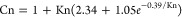
8where  is the Knudsen number, equal to the mean free path of an air molecule λ divided by the radius of a given aerosol. At *T* = 25 °C and ambient pressure of 1 bar, λ ≈ 68 nm.

The immersed bodies (filter fibers and aerosols) are coupled to the fluid according to the difference between the velocity of the structure point of the immersed body **v**_s_(**X**, *t*) and the velocity of the fluid node **v**_f_(**X**, *t*)^78^
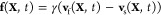
9For the interpolation of the velocities of the fluid nodes to the structure point **X** and for the spreading of the force, we use the Peskin four-point function kernel.^79^ The corresponding force on a given fluid node appears as an external forcing term in the Boltzmann equation.^80,81^

We use OpenFSI coupling package^82^ (see also ref (83)), which efficiently couples a solver for the Lattice-Boltzmann calculations, Palabos,^84^ and a solver for the structure dynamic, LAMMPS.^68^ LAMMPS is used to simulate the Brownian dynamics of the aerosols around the static fiber structures.

To simulate a constant inflow of the aerosols, we keep the simulation box open along the *z* coordinate and perform aerosol deposition in a small region near the inlet. The aerosols are given the same initial velocity as the flow velocity at the inlet. The concentration of the aerosols is kept constant using^85,86^
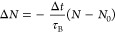
10where Δ*N* is the number of particles to be inserted or deleted in a given time step, τ_B_ is the relaxation time of the chemostat, which is typically on the order of τ_B_ ∼ 100Δ*t*, *N*_0_ is the desired number of particles in the insertion region, while *N* is the current number of particles. Note that finding an optimal placement position of aerosol particles using, e.g., the USHER method^87,88^ is not necessary, but we insert new aerosols at least one lattice unit away from the existing aerosols to improve numerical stability.

As the aerosol particles are dragged by the flow of gas through the filter toward the outlet, some of them may come very close to or even collide with the filter fibers. At each time step in our simulation, we checked the distance between the fiber surface and the aerosol particles. If the distance is equal to or less than the radius of the aerosol particles, then the particles are removed from the simulation, effectively modeling the short-range nature of the interactions between the aerosols and the fiber surface. The process of particle deposition on fiber surfaces and its impact on the known degradation of filter performance are topics of future research.

To calculate the PRE numerically, we measure, after a sufficiently long time, the particle concentration in a small region just above the insertion region *c*_in_ and in a region near the outlet *c*_out_
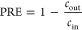
11which is equal to 1, if *c*_out_ = 0, i.e., if all of the particles are captured by the filter, and 0, if *c*_in_ = *c*_out_, i.e., if the filter is very inefficient. In addition, we measure the quality factor *Q*, which takes into account both the particle removal efficiency and the pressure drop^77^
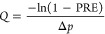
12which is typically measured in kPa^–1^.

To reduce finite size effects, periodic boundary conditions are specified in the lateral (*x* and *y*) dimensions of the filter. At the inlet surface (*z* = 0), a homogeneous velocity *u*_in_ is prescribed, determined from experiments by the ratio of the air volume inflow *q* ≈ 4 L min^–1^ of the air and the surface area of the inlet *A* ≈ 4.9 cm^2^,  m s^–1^. This corresponds to 0.00145 in Lattice-Boltzmann units, which satisfies the required condition of low Mach numbers: . The initial velocity of the particles is also set to *u*_in_. At the outlet, we impose a constant density ρ = 1 (or δρ = 0), which is equivalent to imposing a constant pressure (or δp = 0) at the outlet. As the simulation evolves, a pressure difference is induced between the inlet and outlet, which we refer to as the pressure drop, Δ*p* = *p*_inlet_ – *p*_outlet_. The ranges of the other important simulation parameters used in this work are summarized in Table 1.

**Table 1:** Simulation Parameter Ranges

property	value range
aerosol particle diameter *d*_p_	5–2000 nm
aerosol mass density ρ_p_	2170 kg m^–3^
fiber radius *r*_f_	0.1–1.5 μm
filter thickness Δ*z*	7–36 μm
filter porosity ε	0.80–0.98
inlet volume flux *q*	4 L min^–1^
air density ρ	1.184 kg m^–3^
air kinematic viscosity ν	1.562 × 10^–5^ m^2^ s^–1^
temperature T	25 °C
length scale Δ*x*	0.1 and 1 μm
BGK relaxation time τ	1.0

## 3 Results

### 3.1 Filter Design and Optimization

First, we calculate the pressure drop across a filter in the absence of aerosols. Typical curves of the particle removal efficiency as a function of the particle diameter are presented. Using the particle diameter at which filter efficiency is the lowest, we examine the PRE and the filter quality factor *Q* for various filter geometries, including double-layer filter designs.

#### 3.1.1 Pressure Drop of Single-Layer Filter

We first studied the pressure drop across a filter without aerosols. For easier visualization of the pressure field around the fibers, we have generated a filter with a small (up to 25° w.r.t. the *y* axis) variation of the fiber orientations in the *xy* plane; see Figure 2b. In the remaining results, the orientations of the fibers in the *xy* plane are completely random. Moving from the outlet to the inlet, the pressure drops most in the region of the filter where the fibers are close together; see Figure 2c.

Next we checked the variation of the pressure drop with the inlet velocity. From Figure 2d, one can see that the pressure drop increases linearly as one increases the inlet velocity, which is consistent with the Darcy’s law^89^
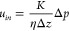
13where *K* is the permeability, η = ρν is the dynamic viscosity of air, and Δ*z* is the thickness of the filter across which we measure the pressure drop Δ*p*. Moreover, the pressure drop increases as one increases the fiber radius *r*_f_; see Figure 2f,g,h for the corresponding filters. We emphasize that the inlet velocity is an important parameter affecting not only the pressure drop but also the PRE curves.^90^ In general, the filtration for small particles dominated by the Brownian motion is more efficient at lower inlet velocities.^77^ Due to the enhanced inertial effect, the particle diameter at which the PRE is the smallest shifts to smaller values at higher inlet velocities.^91^

In this section, the volume inflow is fixed to the value used in experiments, *q* = 4 L min^–1^, which corresponds to the inlet velocity *u*_in_ ≈ 13.6 cm s^–1^. The local fluid velocity *u*_loc_ is higher inside the filter region, where it is inversely proportional to the porosity: . Such an inlet velocity corresponds to low Reynolds number , where 1 μm fiber radius *r*_f_ was chosen as the characteristic length. As a possible reference, we mention the European standard (EN 149:2001+A1:2009),^39^ which specifies a maximum pressure drop in masks at an inhalation flow rate of *q* = 30 L min^–1^ to be 0.6 mbar for FFP1 masks, 0.7 mbar for FFP2 masks, and 1.0 mbar for FFP3 masks.

#### 3.1.2 Particle Removal Efficiency of Single-Layer Filter

To calculate the PRE numerically, we measure the concentrations *c*_in_ and *c*_out_ at a chosen particle radius of about 650 μs and average the last 100 μs of the simulation. The downstream concentration *c*_*out*_ reaches a steady state after about 300 μs. The concentration fluctuates due to both Brownian motion and the random insertion method. We note that since we are neglecting the interactions between aerosols, we can use relatively large aerosol concentrations on the order of 0.1 μm^–3^, which improves the statistics.

To obtain an approximate value for the aerosol particle diameter at which the PRE is the lowest for filters made of micrometer-sized fibers, we calculate the PRE curves as a function of the particle diameter at three different values of fiber radii, *r*_f_ = 0.5 μm, *r*_f_ = 0.75 μm, and *r*_f_ = 1.0 μm; see Figure 2e. The filter thickness is fixed at 24 μm, and the corresponding filter porosities are ε = 0.98, ε = 0.95, and ε = 0.91. The number of fibers is fixed.

One can see that the filter efficiency is high for both small and very large particles. The predominant mechanism for small particles is Brownian motion, which makes the probability of the aerosol colliding with the fiber surface larger as the aerosol particle diameter gets smaller.

**Figure 3:**
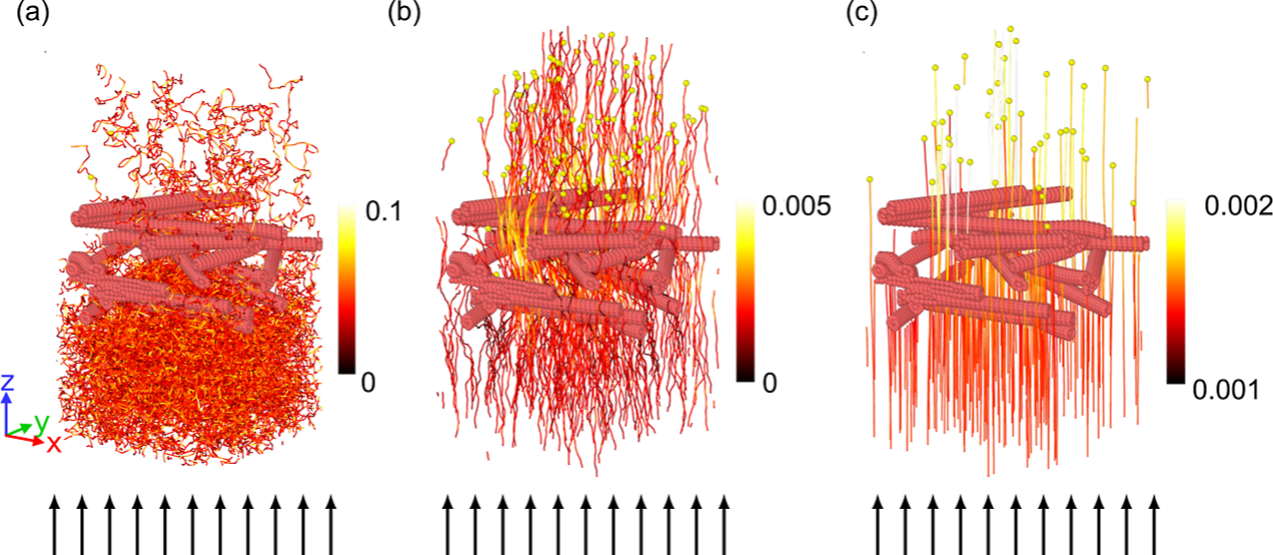
Trajectories of (a) small *d*_p_ = 20 nm, (b) intermediate-sized *d*_p_ = 300 nm, and (c) large *d*_p_ = 2000 nm aerosols through a filter with *r*_f_ = 1 μm. The trajectories of aerosols are color coded according to the magnitudes of their velocities (in Lattice-Boltzmann units). The small arrows represent the direction of the inlet velocity.

Filter materials are also very efficient for larger particles. Due to the large inertia of the larger particles, they do not deviate much from their initial velocity direction and therefore have a high probability of colliding with the fiber surface. Eventually, as one increases the particle diameter, the filter acts like a sieve for the largest particles, and PRE sharply increases to 1. The PRE curve passes through a minimum at about *d*_p_ = 200 nm. At this particle size, Brownian motion and inertial impaction are not efficient removal mechanisms. From here on, this particle diameter has been used in the rest of the simulations. An example of the aerosol trajectories for small *d*_p_ = 20 nm, intermediate-sized *d*_p_ = 300 nm, and large *d*_p_ = 2000 nm aerosols is shown in Figure 3. Due to Brownian motion, the filter is much more efficient for smaller aerosols than for intermediate-sized aerosols. In contrast, very large aerosols pass through only sufficiently large gaps in the filter material.

#### 3.1.3 Effects of Expanding Single-Layer Filter

**Figure 4:**
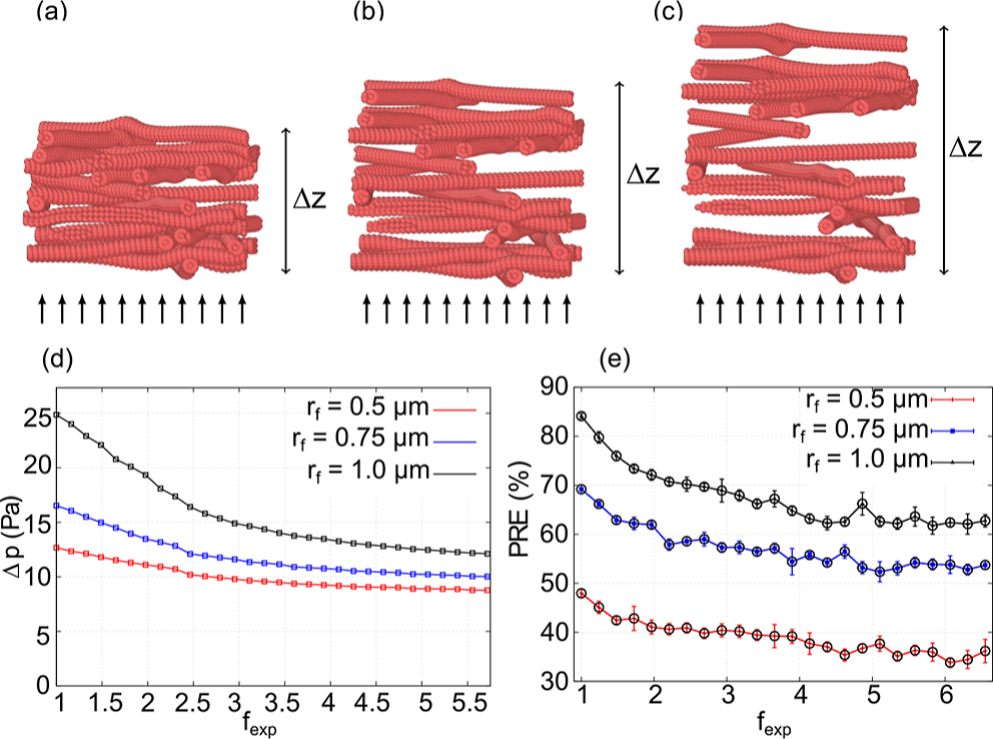
Examples of filter morphologies at three different filter expansion factors: (a) *f*_exp_ = 1, (b) *f*_exp_ = 1.44, and (c) *f*_exp_ = 1.88. The small arrows represent the direction of the inlet velocity. (d) Pressure drop across the filter as a function of expansion factor *f*_exp_ at three different fiber radii. The number of fibers is kept constant as is the random seed with which they are generated. (e) PRE as a function of expansion factor *f*_exp_ at three different fiber radii *r*_f_. The particle diameter is fixed at 200 nm.

To study the effects of expanding a filter, we rescale the initial positions of the fibers along the *z* coordinate; see Figure 4a–c. The resulting filter thickness is Δ*z* = *f*_exp_Δ*z*^0^, where *f*_exp_ > 1 is the expansion factor, and Δ*z*^0^ is the filter thickness of the initial configuration, which in our case is equal to 24 μm. Experimentally, this can be achieved by adding, e.g., polystyrene spacer beads between the individual layers of electrospun fibers or, in principle, by adding a highly porous support material between the layers. The number of fibers *N*_f_ is kept constant, and the particle diameter is fixed at *d*_p_ = 200 nm. The effects of expanding a filter in which the solid content is held constant have notable practical relevance. More specifically, an expanded filter is more comfortable to wear due to the lower pressure drop and still provides a reasonably good PRE.

Steady-state pressure drop as a function of the filter thickness, keeping the number of fibers constant, is shown in Figure 4d. A decrease in the pressure drop with increasing expansion factor *f*_exp_ of the filter is observed, which is expected since expanding a filter increases the local porosity of the filter. The decrease is more pronounced for higher filter radii and low filter thicknesses. This means that the variation of the pressure drop is an important factor when studying the quality factor at different filter thicknesses. This decrease can be qualitatively explained by Davies’ empirical formula for the pressure drop^92^
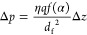
14where *d*_f_ = 2*r*_f_ is the fiber diameter, α = 1 – ε is the solid volume fraction, and *f*(α) = 64α^1.5^(1 + 56α^3^) is an empirical function valid in the range 0.006 < α < 0.5. When expanding a filter, the solid volume fraction decreases inversely with *f*_exp_, , where α_0_ is the solid volume fraction of the initial configuration. The thickness of the filter increases linearly with *f*_exp_, Δ*z* = Δ*z*^0^*f*_exp_. The resulting pressure drop therefore decreases, with the leading term inversely proportional to the square root of the expansion factor
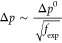
15where Δ*p*^0^ is the pressure drop of the initial configuration. In our case, the decrease in the pressure drop is more gradual and seems to approach a constant value; see Figure 4d. This is because some of the fibers in our computer-generated filters initially have a very similar *z* coordinate, which means that the local solid volume fraction does not change drastically with *f*_exp_.

As one expands such a filter, the particle removal efficiency decreases; see Figure 4e. The reason for this decrease is the higher overall porosity. When a filter is stretched, the spacing between the fibers increases, reducing the magnitude of the local velocity field due to the higher porosity ε. Consequently, a decrease in velocity reduces the contribution of inertial impaction mechanism to the PRE, which, in the single fiber efficiency theory, is proportional to the Stokes number Stk^77^
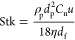
16where *u* is the velocity of the carrier fluid. Similarly, the paths traveled by aerosol particles are on average more convoluted in compressed filters than in expanded ones. Such paths are associated with a higher tortuosity, i.e., the ratio between the actual flow path distance of a particle and the straight line distance between the ends of the flow path. A higher tortuosity is well-known to increase the particle removal efficiency in porous filters.^93^

**Figure 5:**
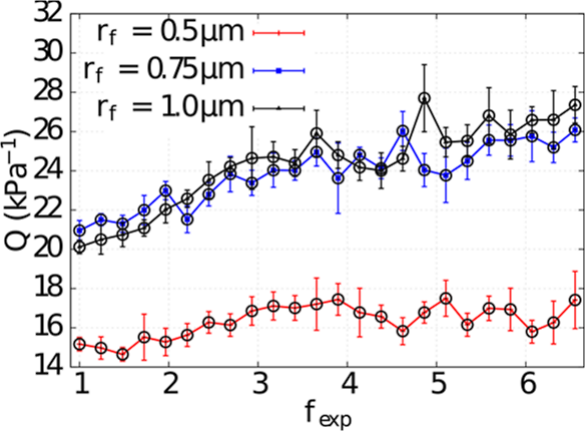
Quality factor as a function of the expansion factor *f*_exp_ at three different fiber radii *r*_f_.

As expected, the PRE is higher for larger fiber diameters, as can be seen in Figure 4e. This should not be confused with the result from the single fiber efficiency theory,^77,94^ where the porosity is held constant and the corresponding PRE decreases for larger fiber diameters. On the other hand, the quality factor of the filter, which also takes into account the pressure drop, is higher for larger filter thicknesses; see Figure 5. Moreover, we see that the quality factor of the filter with *r*_f_ = 0.75 μm is similar to the quality factor of the filter with *r*_f_ = 1.0 μm despite the lower PRE for the same thickness.

#### 3.1.4 Double-Layer Filter

Double-layer filters consist of a layer of thick micron-sized fibers and a layer of thin electrospun PVDF-HFP nanofibers with diameters between 80 and 300 nm. Experimentally, one can control the thickness of the layers and, to some extent, the diameter of the fibers.

**Figure 6:**
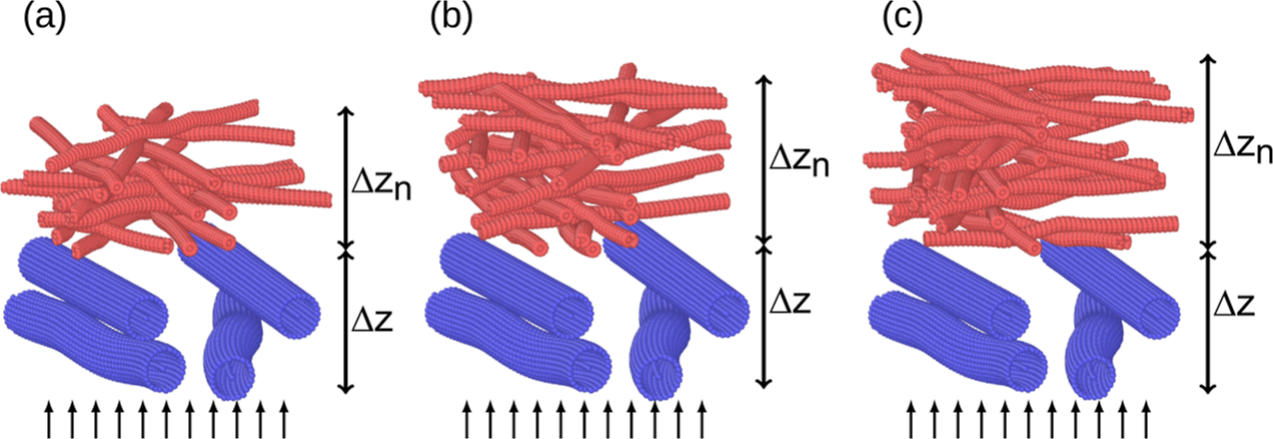
Examples of double-layered filters at three different widths of the nanofiber layer (red): (a) Δ*z*_n_ = 1.8 μm, (b) Δ*z*_n_ = 2.4 μm, (c) Δ*z*_n_ = 3.1 μm. The thickness of the layer of 1 μm thick fibers (blue) is set to Δ*z* ≈ 2.1 μm. The small arrows represent the direction of the inlet velocity.

In Figure 6, we show the computer-generated model of a double-layer filter at different nanofiber layer widths Δ*z*_n_. We fix the thickness of the layer consisting of *d*_f_ = 1 μm thick fibers to Δ*z* ≈ 2.1 μm, which corresponds to about two fiber diameters. Then, we vary the thickness of the layer consisting of *d*_f_ = 200 nm fibers from Δ*z*_n_ = 0 to Δ*z*_n_ ≈ 5.5 μm. The porosity of the thick layer is ε ≈ 0.83, while the porosity of the thin layer is varied in a range between ε ≈ 0.93 and ε ≈ 0.98.

**Figure 7:**
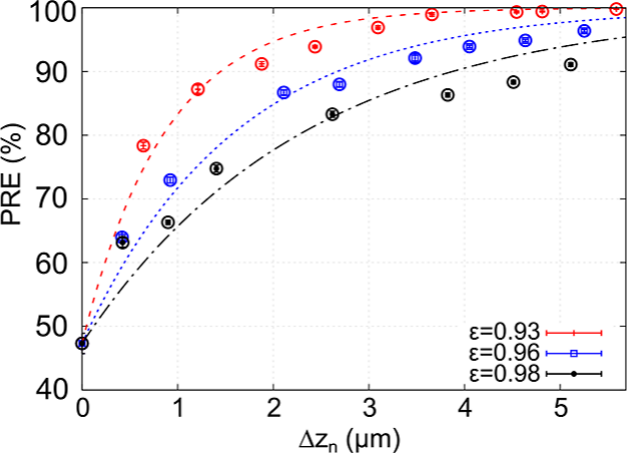
PRE of the filter for the *d*_p_ = 200 nm aerosol particle as a function of the thickness of the electrospun layer Δ*z*_n_. The thickness of the layer of thick micrometer-sized fibers is fixed at Δ*z* ≈ 2.2 μm. The numerical data are fit to eq 17.

**Figure 8:**
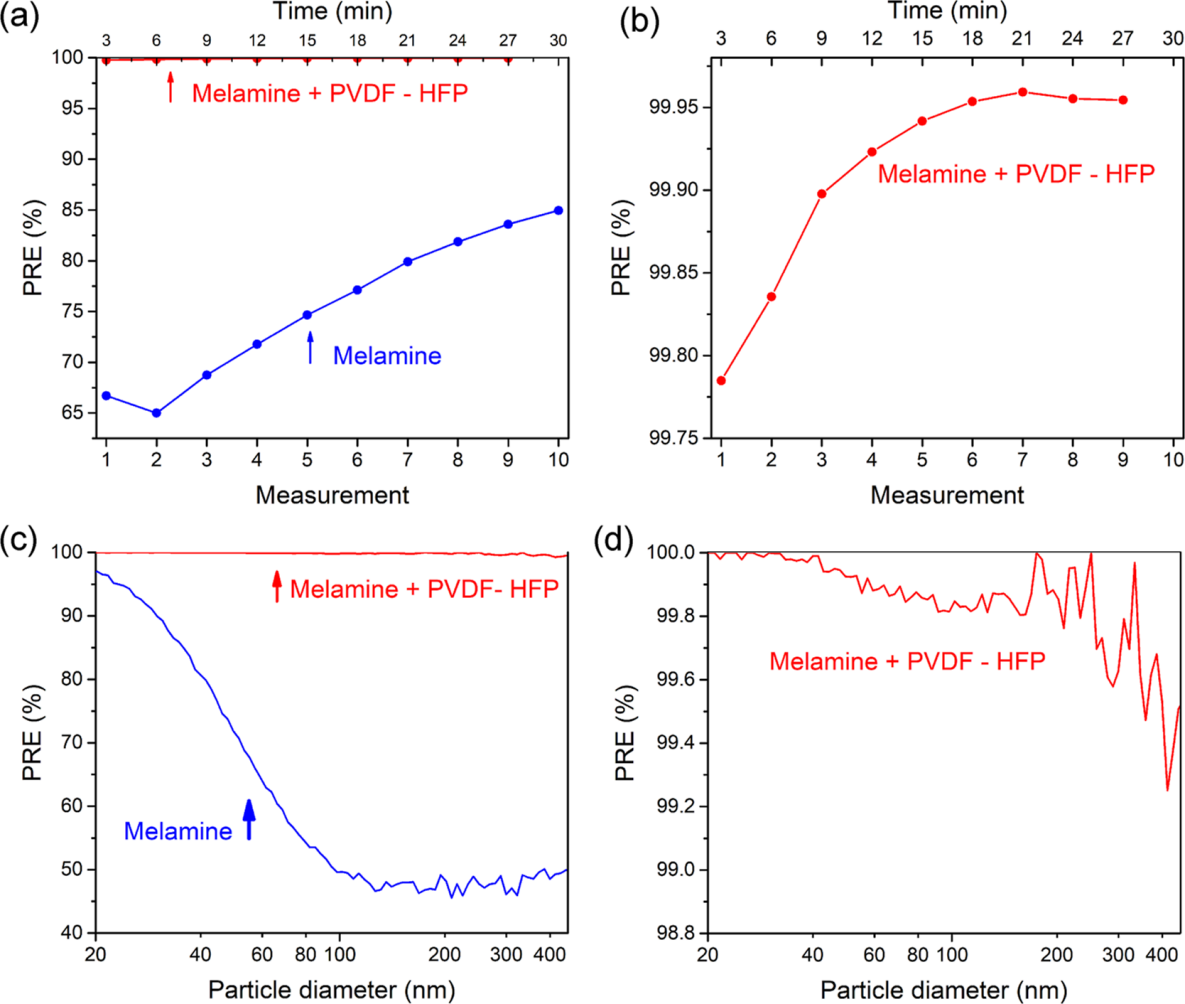
Overall PRE (see eq 1) of the filter as a function of the measurement number: (a) melamine without PVDF-HFP fibers; (b) melamine with PVDF-HFP fibers. PRE of the filter as a function of the particle diameter *d*_p_ (see eq 2): (c) melamine without PVDF-HFP fibers; (d) melamine with PVDF-HFP fibers. The PRE data is acquired in the third measurement, i.e., between the sixth and the ninth minute after the start of the measurements.

We show that adding a second thin layer of (electrospun) fibers significantly improves the PRE, as shown in Figure 7. Moreover, we find that the PRE increases to 1 exponentially as one increases the thickness of the nanofiber layer. This is in good agreement with the theoretical result from the single fiber efficiency theory: , where *g* depends on several parameters, such as the inlet velocity, the fiber radius, the fiber distribution within the filter, and the porosity.^77^ In the double-layer filter, we vary the thickness of the thin fiber layer, while the thickness of the layer of thick fibers is fixed. We fit the numerical results to the following PRE dependence
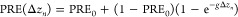
17where PRE_0_ is the PRE of the layer consisting of thick fibers. The fit function in eq 17 is a simple modification of the PRE dependence on filter thickness from the single fiber efficiency theory.^77^ For the fit parameter *g*, we extract the values *g* = (1.15 ± 0.07) μm^–1^, *g* = (0.62 ± 0.04) μm^–1^, and *g* = (0.43 ± 0.04) μm^–1^, corresponding to filters with the porosities ε = 0.93, ε = 0.96, and ε = 0.98, respectively. The significant increase of the PRE for a double-layer filter is also demonstrated by our experiments, Figure 8.

As expected, the efficiency increases when one decreases the porosity of the nanofiber layer. However, better efficiency for a nanofiber layer with lower porosity is accompanied by a higher pressure drop. Consequently, for the selected nanofiber filter porosities, the nanofiber layer with ε = 0.98 shows an optimal performance in terms of the quality factor *Q* (Equation 12) in the chosen range of nanofiber layer thickness (0.0–5.5 μm).

### 3.2 Experiments

#### 3.2.1 Pressure Drop

The pressure drop is measured in accordance with the EN 14683 standard using the Air Permeability Tester FX 3340 minAir. The air flow rate is 8 L min^–1^. Area of the samples is 4.9 cm^2^. Grammage of the samples is 60 gsm, and it was the same before and after the PVDF-HFP spinning. The pressure drop (Δ*p*) is calculated per surface unit.

The pressure drop measured on the smartMELAMINE without PVDF-HFP nanofibers is 28.6 Pa cm^–2^. The pressure drop measured on the smartMELAMINE covered with electrospun PVDF-HFP nanofibers is 38.8 Pa cm^–2^. The results show that the electrospun PVDF-HFP nanofiber increases the pressure drop of the smartMELAMINE nonwoven textile by approximately 35%.

#### 3.2.2 Particle Removal Efficiency

The particle removal efficiency was measured for single-layer and double-layer fibers; see Figure 8. Overall PRE of melamine fibers without PVDF-HFP fibers was around 70% and increased with time due to the accumulation of particles on the filter; see Figure 8a. Within a span of 24 min (corresponding to eight 3 min long sequential measurements), the overall PRE increased to 84%. This filter primarily removes particles smaller than 100 nm, while PRE for larger particles is within a range of 50–60%; see Figure 8c. It can be concluded that the process of filtration for the chosen aerosol size range (13–572.5 nm) is mainly diffusive.

The overall PRE of melamine fibers covered with electrospun PVDF-HFP fibers was 99.9%. Within a 20 minute time frame, the PRE saturated at about 99.95%; see Figure 8b. As a function of particle diameter, this filter primarily removes all particles with diameters below 500 nm; see Figure 8d. The lowest efficiency exceeds 99% for all particles in the measured range. The quality factor for the material without the electrospun PVDF-HFP nanofibers is *Q* = 8.6 kPa^–1^, which is more than four times smaller compared to the quality factor of the material with electrospun PVDF-HFP fibers, which is *Q* = 36.3 kPa^–1^.

Our experimental results show that adding a thin nanofibrous layer of PVDF-HFP on top of a highly permeable melamine layer increases its particle removal efficiency to values above 99% over the entire range of particle sizes. This was also qualitatively demonstrated by our numerical simulations, see Figure 7, where the PRE for a 200 nm aerosol particle increased from 50% to over 90% when only a 2 μm thick nanofiber layer was added. Compared to the uncoated melamine filter, which does not filter well the larger particles due to a large opening between the fibers, the double-layer filter (melamine coated with PVDF-HFP) exhibits high filtration efficiency already in the first 3 min cycle of the test. The openings between the PVDF-HFP fibers are at least 2 orders of magnitude smaller, and the diffusion mode of filtration is combined with the impaction and the interception mode. It is noteworthy that the single-layer (melamine fibers only) filters did not filter as efficiently as the double-layer filters even when the tested material loaded the filter. This can be explained by a three-dimensional loading of small particles of the standard powder, which clustered in the spacings between the fibers but did not clog them. Conversely, in the double-layer filter, they formed a less dense and thick flat coating (Figure 1g).

## 4 Discussion and Conclusions

Two possible filter design changes that increase the performance of mechanical fibrous filters, in terms of either particle removal efficiency or quality factor, were investigated. Methodologically, we combined the Lattice-Boltzmann method for modeling gas flow, the immersed boundary method for coupling the flow to the fiber structures, and the Langevin dynamics of the aerosols. The results were compared and tested against experiments.

The results show that an extension of the filter—that is having less densely packed fibers with more interstitial space (see Figure 4a–c)—increases its performance in terms of the quality factor. The reason for this increase is a smaller pressure drop. The particle removal efficiency decreases somewhat as one expands the filter, since the paths from the inlet to the outlet are on average less tortuous than those for compressed and less porous filters. Additionally, we have also investigated the impact of adding a nanofiber layer with diameters in the nanometer range on top of a highly permeable layer of micrometer-sized fibers, both numerically and experimentally. Our simulations show that adding just a thin, highly porous (ε between 0.93 and 0.98) layer with a thickness on the order of 2 μm increases the PRE by 30–40%. The motivation for such a two-layer design is to enable the use of a base layer made out of thermally stable melamine, which is also chemically stable and UV resistant, offering an advantage over other materials, as it can be sterilized without losing its properties. It also has fire-retardant properties, which are essential for protective equipment in an oxygen-enriched atmosphere, such as hospitals.^95^ Furthermore, the high temperature stability allows thermal disinfection of the used respiratory masks, which could significantly reduce the present environmental burden.^96^ Indeed, when thin electrospun fibers made from inert PVDF-HFP fibers are used as an overlayer on thicker melamine fibers, PRE improves greatly from 70 to 99.9%. The pressure drop increases by 35% from 0.29 to 0.39 mbar but remains below the limit value for both FFP1 masks (0.6 mbar) and type IIR surgical masks (0.49 mbar).

As a side note, we should mention that both PVDF-HFP-based nanofibers and melamine microfibers are stable over a wide temperature range. The PVDF-HFP has a glass transition temperature of about −35 °C and can withstand temperatures up to 150 °C, while melamine microfibers are stable up to 240 °C. Such thermal stability of these materials makes them viable candidates for hot gas filtration devices.^97^ Although the effects of temperature on the filtration efficiency were not considered in this work, we expect greater particle removal efficiency for small aerosols due to the enhanced Brownian motion. On the other hand, we expect a larger pressure drop at higher temperatures relative to the higher air viscosity. In terms of the mechanical stability of nanofibers and melamine microfibers, we do not expect much change within a reasonable temperature range (−10 to 40 °C).

As a future perspective, we mention the effects of electrostatic interaction^98,99^ between aerosols and fibers since nanofibers made of a β-phase of PVDF-HFP^100^ have nonzero polarization. More specifically, the polarization distribution, i.e., its orientation along the nanofiber, is an important possible additional mechanism that could, in principle, be tuned for better filter performance. Another perspective is to include the filter degradation effects, where the captured aerosol particles build up on the fibers and consequently increase the pressure drop. In the current model, these effects are neglected and the pressure drop saturates at a finite value.

## Supporting Information Available

Scheme of the simulation method workflow for the computational filter design and calculation of the filter efficiency (PDF)
