# Supplementary material for: Single-Layer and Double-Layer Filtration Materials Based on Polyvinylidene Fluoride-Co-hexafluoropropylene Nanofibers Coated on Melamine Microfibers
Source: ACS Appl Nano Mater. 2023 Aug 22;6(17):15807–19. doi: 10.1021/acsanm.3c02592 (PMC10496027; doi:10.1021/acsanm.3c02592)
Supplement: Supplementary file 1 — an3c02592_si_001.pdf [file an3c02592_si_001.pdf]

# Supporting Information: Single-Layer and Double-Layer Filtration Materials Based on Polyvinylidene Fluoride-Co-Hexafluoropropylene Nanofibers Coated on Melamine Microfibers

Tilen Potisk,[*,†,‡] Maja Remškar,[¶] Luka Pirker,[¶,§] Gregor Filipič,[¶] Igor Mihelič,[‖] Marjan Ješelnik,[‖] Urban Čoko,[†,‡] and Miha Ravnik[‡,¶]

†*Laboratory for Molecular Modeling, National Institute of Chemistry, SI-1001 Ljubljana, Slovenia*

‡*Faculty of Mathematics and Physics, University of Ljubljana, SI-1001 Ljubljana, Slovenia*

¶*Jožef Stefan Institute*

*SI-1000 Ljubljana, Slovenia*

§*J. Heyrovsky Institute of Physical Chemistry*

*Czech Academy of Sciences*

*Prague 8, Czech Republic*

‖*MELAMIN d.d.*

*SI-1330 Kočevje, Slovenia*

E-mail: tilen.potisk@ki.si

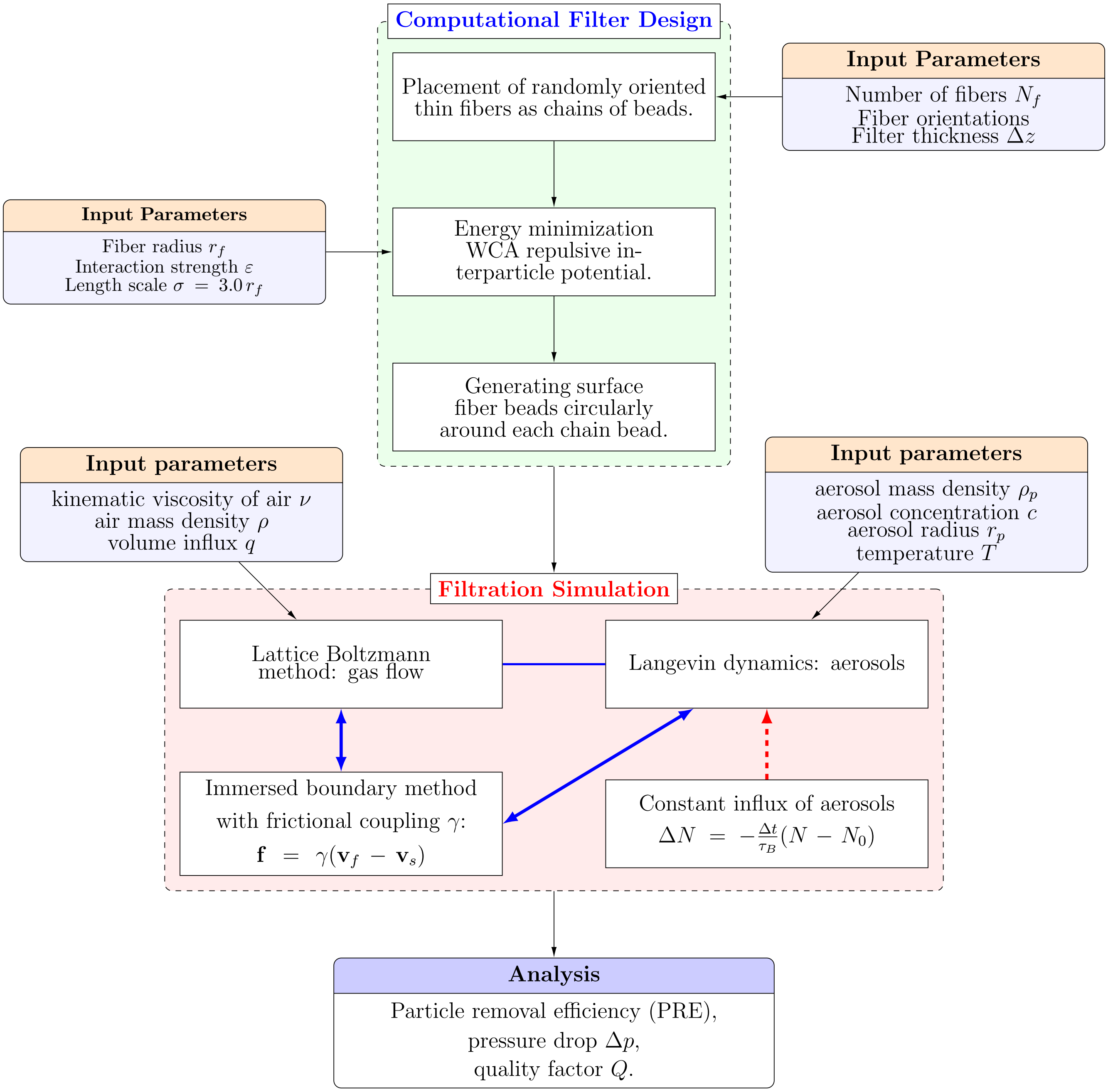


Figure S1: Flowchart of the simulation method.
